# Expression and Prognostic Value of Melanoma-Associated Antigen D2 in Gliomas

**DOI:** 10.3390/brainsci12080986

**Published:** 2022-07-26

**Authors:** Jun Yan, Shenyu Li, Cameron Lenahan, Hainan Chen, Jing Wen, Qianrong Huang, Qian Jiang, Fangzhou Guo, Teng Deng, Ligen Mo

**Affiliations:** 1 Department of Neurosurgery, Guangxi Medical University Cancer Hospital, Nanning 530021, China; yanjun@gxmu.edu.cn (J.Y.); h2013012@sr.gxmu.edu.cn (H.C.); huangqianrong@gxmu.edu.cn (Q.H.); 20185010599@sr.gxmu.edu.cn (Q.J.); guofangzhou@gxmu.edu.cn (F.G.); dengteng@gxmu.edu.cn (T.D.); 2 Department of Neurosurgery, Second Affiliated Hospital of Guilin Medical University, Guilin 541100, China; swlishenyu@glmc.edu.cn; 3 Department of Biomedical Sciences, Burrell College of Osteopathic Medicine, Las Cruces, NM 88001, USA; cameron.lenahan@burrell.edu; 4 Department of Rheumatism, First Affiliated Hospital of Guangxi Medical University, Nanning 530021, China; wenjing@sr.gxmu.edu.cn

**Keywords:** melanoma-associated antigen-D2, CDKN1A, glioma, proliferation, prognosis

## Abstract

Introduction: The melanoma-associated antigen D2 (MAGED2) is one of the melanoma-associated antigen family members. It is commonly overexpressed in a variety of malignancies. However, the mechanism and function of MAGED2 in glioma remain unknown. Methods: The MAGED2 expression level and the correlations between clinical characteristics were analyzed with the data from the CGGA and TCGA datasets. MAGED2 expression in 98 glioma tissues was measured using RT-qPCR, Western blot, and immunohistochemistry. CCK-8, colony formation, and EdU assays were used to assess the effect of MAGED2 on U251-MG cell proliferation. Flow cytometry was used to track changes in the cell cycle and cell apoptosis following plasmid transfection with CRISPRi. Results: MAGED2 was shown to be highly expressed in glioma tissues, and high MAGED2 expression predicted poor prognosis. Furthermore, MAGED2 knockdown significantly inhibited the proliferation of U251-MG cells by preventing cell cycle arrest at the G0/G1 phase and triggering apoptosis. In line with in vitro findings, the results of the xenograft experiment and immunohistochemistry also showed that MAGED2 suppression inhibited tumor development and decreased Ki-67 expression levels. Conclusions: MAGED2 may be a possible biomarker for glioma and an important prognostic factor for glioma patients.

## 1. Introduction

Malignant glioma remains the most frequent and severe primary brain tumor, with substantial morbidity and fatality rates [1]. Glioma can be treated using a variety of methods, including surgery, radiation, and chemotherapy. However, the prognosis for glioma patients, particularly glioblastoma (GBM), remains poor as a result of the high tumor recurrence rate [2]. Glioma is characterized by rapid cell proliferation. This dismal prognosis is largely due to the proliferative nature of malignant glioma [3]. At present, the molecular mechanisms underlying the proliferation of gliomas are gradually becoming known; consequently, it is vital that a more thorough understanding of the biological and molecular mechanisms underpinning glioma formation and progression is procured to develop more effective therapies [4,5,6,7].

The melanoma-associated antigen D2 (MAGED2) is a member of the melanoma-associated antigen family, which is consistent with type-I genes (MAGE-A, -B, and -C) and type-II genes (MAGE-D, -E, -F, -G, and -H) [8]. The overexpression of melanoma-associated antigens (MAGEs) is associated with a poor prognosis in glioma patients [9,10,11]. One study used a database to search for type-II genes and came to the conclusion that the expression of type-II genes is related to significant clinical and molecular characteristics in gliomas [12]. In normal conditions, MAGED2 is present in the cytoplasm, nucleoplasm, and nucleoli. However, MAGED2 is a dynamic protein whose shuttling properties could suggest a role in cell cycle regulation [13]. MAGED2 has been identified as being critical for transcriptional regulation [14], epigenetic alteration [12], and cell development and differentiation [15]. Furthermore, numerous studies have shown that MAGED2 is a tumor-specific antigen that plays an important role in tumor progression and metastasis, including hepatocellular carcinoma [16], gastric cancer [17], and lymphoma [18]. As a result, MAGED2 is expected to become a potential cancer target, and may play an important role in tumor progression. However, the precise function and mechanism of MAGED2 in glioma is still unknown. The goal of current research aims to investigate the MAGED2 gene’s potential as a cancer-associated tumor marker, which might serve as a beneficial prognostic biomarker and immunotherapeutic target for glioma.

## 2. Materials and Methods

### 2.1. Bioinformatic Analyses

On the basis of the Cancer Genome Atlas-Cancer Genome (TCGA database, https://cancergenome.nih.gov/ (accessed on 15 January 2022)), prognostic data from glioblastoma (GBM) patients and low-grade glioma (LGG) patients were obtained from the OncoLnc database (https://www.oncolnc.org/ (accessed on 10 February 2022)). Furthermore, overall survival (OS) has been calculated in days from the time of diagnosis to the time of death. Additionally, the mRNA expression of MAGED2 in a variety of glioma types was collected from the Oncomine dataset (https://www.oncomine.org (accessed on 5 January 2022)) [19]. The MAGED2 mRNA expression in various grades of glioma was obtained from the Chinese Glioma Genome Atlas (CGGA database, https://www.cgga.org.cn (accessed on 12 February 2022)). The information on IDH1 mutation and MGMT expression in LGG and GBM was received from the UCSC Xena browser, which is based on the TCGA database (https://xenabrowser.net (accessed on 20 June 2022)) [20]. The relationship between MAGED2 expression and IDH1 mutation (or MGMT expression) was assessed. Two high-expression and low-expression groups were created based on the median level of MGMT expression. The goal of immunohistochemical staining is to determine where the MAGED2 protein is found within the cell. The MAGED2 protein was mostly found in the cytoplasm and nucleus of the cells [13]. The immunohistochemistry staining data of MAGED2 in glioma and normal tissues were obtained from the Human Protein Atlas (www.proteinatlas.org (accessed on 20 June 2022)) and were used to calculate the overall percentage of positive cells for MAGED2 protein.

### 2.2. Patient Characteristics and Tissue Specimens

The current study included the evaluation of 98 cases of glioma from patients ranging in age from 19 to 82 years (median age, 39 years) at the time of diagnosis. There were 59 men and 39 females among these patients. In addition, 12 samples of normal brain biopsy tissue collected from epileptic resections were included as controls in the research. Pre-operative radiation or chemotherapy was not administered to any of the patients. Tumor tissues were obtained sequentially from patients at Guangxi Medical University Cancer Hospital (Nanning, China) between September 2016 and June 2021, and were evaluated after receiving permission to conduct research from the Guangxi Medical University Ethics Committee. All patients gave their informed consent to participate in this study. The retrospective database was examined for demographics, tumor size, WHO Classification, Ki-67 expression, IDH1/2, and MGMT. A telephone interview was conducted with 98 patients (Table 1). Every 3–6 months, post-operative follow-up exams were performed, which included a physical examination and a computed tomography scan. Depending on the tumor status, tumor grade, and patient condition, a treatment approach was selected from the options of surgery, adjuvant radiation, and chemotherapy. Prior to the examination, tissue specimens were stored in liquid nitrogen at −80 °C. The 2021 WHO Classification of Tumors of the Central Nervous System was used for histological analysis and tumor staging [21].

### 2.3. Cell Lines and Cell Cultures

The Chinese Academy of Sciences (Shanghai, China) provided the human malignant glioma cell line, U251-MG. The cell lines were grown in a complete medium made up of Dulbecco’s modified Eagle’s medium (DMEM) supplemented with 10% fetal bovine serum (FBS, Hyclone, Logan, UT, USA) and penicillin/streptomycin. The cells were grown at 37 °C in a humidified environment with 5% CO_2_.

### 2.4. CRISPR Interference (CRISPRi) of MAGED2

CRISPR/Cas9 KO Plasmid Transfection was achieved as described previously according to [22]. Santa Cruz Biotechnology (Dallas, TX, USA) provided the MAGE-D2 CRISPR/Cas9 KO Plasmid (#sc-405573). For CRISPRi experiments, plasmid-containing dead Cas9 (dCas9), MAGED2 domain, and single guide RNA (sgRNA) were constructed by Santa Cruz Biotechnology (Dallas, TX, USA). Designed sgRNAs were cloned into pSpCas9n(BB)-2A-Puro (PX462) V2.0 using the following sequences: MAGED2 CRISPRi-sgRNA-1f: 5′-GACTTTACCCGAGCGCTTGA-3′; MAGED2 CRISPRi-sgRNA-1r: 5′-TGCAGGTCTAACTCGCTTCC-3′; MAGED2 CRISPRi-sgRNA-2f: 5′-GAAACAGAATGCTGACCCGC-3′; MAGED2 CRISPRi-sgRNA-2r: 5′-ATGTCTGACACAAGCGAGAG-3′; MAGED2 CRISPRi-sgRNA-3f: 5′-GAATCAGGATACTCGGCCCA-3′; and MAGED2 CRISPRi-sgRNA-3r: 5′-TCGTCTGGTCTTTAGCCAAA-3′. The resulting plasmids, PX459_sgMAGED2_1, PX459_sg MAGED2_2, and PX459_sg MAGED2_3, were transfected pairwise into cells using Lipofectamine 2000 (DNA:reagent-ratio 1:1 for U251-MG) according to the manufacturer’s protocols. Three–five days hereafter, the cells were put under puromycin (Santa Cruz Biotechnology, Inc., Dallas, TX, USA) selection (4 µg/mL for U251-MG) for 48 h and finally seeded as single cells into 96-well plates for expansion. The cells were examined under a fluorescence microscope 72 h after transfection, and the knockdown effectiveness of MAGED2 CRISPR was confirmed by qRT-PCR and Western blot. Meanwhile, cells that had been transfected with scrambled CRISPR were used as a control.

### 2.5. CDKN1A siRNA

Thermo Fisher Scientific Inc. (Waltham, MA, USA) provided the CDKN1A siRNA, which was transfected into U251-MG glioma cells using Lipofectamine 2000 according to the manufacturer’s instructions [23]. Meanwhile, cells that had been transfected with a fluorescent siRNA with no homology to the target gene (CDKN1A) sequence were used as a negative control. U251-MG cells were plated at a density of 1 × 10^5^ per well of a 6-well plate for plasmid transfection. The cells were examined under a fluorescence microscope 72 h after transfection.

### 2.6. Quantitative Real-Time Polymerase Chain Reaction (qRT-PCR)

qRT-PCR was conducted as previously reported [9]. TRIzol reagent (Thermo Fisher Scientific, Waltham, MA, USA, #15596026) was used to extract the total RNA from glioma tissues and normal brain specimens. Then, using oligo(dT) and the RevertAidTM First Strand cDNA Synthesis Kit (ThermoScientific, Waltham, MA, USA, #K1622), the cDNA for PCR assay was prepared from 2 µg total RNA. The semi-quantitative RT-PCR and quantitative RT-PCR (qRT-PCR) were performed using a DNA thermocycler Veriti (Life Technologies, Carlsbad, CA, USA) and CFX96 Touch (BioRad, Hercules, CA, USA), respectively. Primers for MAGED2 [24], Cyclin-dependent kinase inhibitor 1A (CDKN1A) [25], and glyceraldehyde-3-phosphate dehydrogenase (GAPDH) [26] were obtained from Applied Biosystems (Thermo Fisher Scientific, Inc., Waltham, MA, USA). The forward primer sequence for MAGED2 was 5′-CCAGCAAGATGAAAGTCCTCA-3′ and the reverse primer sequence was 5′-TCCATCGCCTCTCGGTACT-3′; the forward primer sequence for CDKN1A was 5′-TGTCTTGTACCCTTGTGCCT-3′ and the reverse primer sequence was 5′-GGCGTTTGGAGTGGTAGAAA-3′; and the forward primer sequence for GAPDH was 5′-GAGCCCGCAGCCTCCCGCTT-3′ and the reverse primer sequence was 5′-CCCGCGGCCATCACGCCACAG-3′. The 2^−ΔΔCT^ method was used to quantify MAGED2 mRNA expression levels, which were then normalized by the GAPDH mRNA expression level.

### 2.7. Immunohistochemistry (IHC)

IHC was conducted in accordance with previous research [9]. The tissues are formalin-fixed for 24 h at room temperature before being paraffin-embedded, and then cut into 4 µm-thick slices. Sections of paraffin-embedded tissues were dried for 1 h at 60 °C, dewaxed with xylene, and rehydrated with a declining sequence of alcohols (95, 80, 70, and 50%, *v*/*v*). Next, the slices were incubated overnight at 4 °C with the primary antibody (anti-MAGED2, PA5-113520,1:200; anti-Ki-67, MA5-14520, 1:200; anti-CDKN1A, TA808128, 1:150, Thermo Fisher Scientific, Waltham, MA, USA). Subsequently, the slices were then treated for 20 min at room temperature with an HRP-conjugated secondary antibody (1:500). Finally, every antibody staining was developed for 5 min at room temperature using 3,3′-diaminobenzidine (DAB; Fuzhou Maixin Biotech Co., Ltd., Fuzhou, China) as the chromogen and counterstained with hematoxylin. A known glioma tissue segment with MAGED2-positive expression served as a positive control, whereas the omission of the primary antibody served as a negative control. According to the staining characteristics of MAGED2 protein, MAGED2 is present in the cytoplasm and nucleus [13]. The total of the staining intensity and positive cell rate scores was used to get the final protein expression score, as previously described [9]. Two independent pathologists used a blind technique to determine the presence of positive immunoreactivity. At least five independent foci of neoplastic infiltration in each tissue specimen were observed using an optical microscope (Nikon Corporation, Tokyo, Japan) at magnifications of ×100 and ×200.

### 2.8. Western Blot Analysis

Western blot was conducted as previously reported [27]. The total protein of cells or tissues was extracted. The cells were harvested 72 h after transfection, and RIPA lysis solution was added for 30 min at 4 °C to obtain cell lysates. The concentration of protein was then determined using a BCA Protein Assay Kit (Pierce, Rockford, IL, USA). For each treatment, an equivalent quantity of total protein was separated using 12.5% SDS-PAGE and then transferred to PVDF membranes. The membrane was blocked and incubated overnight at 4 °C with the primary antibodies listed below: anti-MAGED2 (1:1000, PA5-113520, sc-130443), anti-CDKN1A (1:2000, TA808128), and anti-GAPDH antibodies (1:5000, ab8245). GAPDH was used as an internal loading control. The respective secondary antibodies (1:3000, Santa Cruz (Dallas, TX, USA); 1:5000, Abcam, Cambridge, UK) were incubated for 2 h at room temperature.

### 2.9. Cell Counting Kit-8 (CCK-8) Assay

Cell counting kit-8 (Abcam, Cambridge, UK) was applied to assess cell proliferation. U251-MG cells were collected in the exponential phase, infected with MAGED2 CRISPR or Scramble CRISPR, injected at a density of 4 × 10^3^ cells/well, and processed in 96-well plates. Ten μL of CCK-8 solution was added to each well after 0, 24, 48, 72, 96, and 120 h. After 2 h, the absorbance at 450 nm was measured using a microplate reader (Thermo Fisher Scientific, Inc., Waltham, MA, USA). The experiments were independently performed three times, each in three copies.

### 2.10. Colony Formation Assay

A colony formation assay was conducted as previously reported [28]. U251-MG cells were estimated and injected onto 6-well plates at a density of 300 cells/well. Three weeks after the cell was cultured at 37 °C, visible colonies were fixed with 4% paraformaldehyde for 30 min and stained with 0.1% crystal violet for another 30 min. Finally, the efficiency of the colony formation was calculated as the number of colonies (diameter > 0.5 mm) per plated cell × 100%.

### 2.11. 5-Ethynyl-2′-deoxyuridine (EdU) Proliferation Assay

U251-MG cells were estimated and injected onto 24-well plates with a density of 1 × 10^4^ cells/well after infection with MAGED2 CRISPR or Scramble CRISPR. The cells were exposed to 50 M EdU (Invitrogen, Cat. no. A10044) for 2.5 h after incubation. Then, they were fixed, permeabilized, and stained with the Apollo^®^ reaction cocktail for EdU and Hoechst 33,342 (5 g/mL) for U251-MG cell nuclei 48 h later. The ratio of EdU-stained cells (with red fluorescence) to Hoechst-stained cells (with blue fluorescence) was used to evaluate the cell proliferation activity. After, samples were examined under a fluorescent microscope.

### 2.12. Flow Cytometry

Flow cytometry was conducted as previously reported [29]. Before being harvested, Glioma U251-MG cells were infected with MAGED2 CRISPR or Scramble CRISPR and cultured at 37 °C. After, the cells were rinsed twice in phosphate-buffered saline (PBS) and fixed in 75% ice-cold ethanol. Next, the cells were stained with the Cell Cycle Staining Kit (Multi Sciences, Hangzhou, China) and incubated in the dark for 30 min according to the manufacturer’s instructions. U251-MG cells were also collected by trypsinization and treated with FITC-conjugated Annexin V and PI according to the manufacturer’s instructions to assess the influence of MAGED2 CRISPR infection on cell apoptosis (Keygen Biotech, Nanjing, Jiangsu, China). Finally, flow cytometry was used to examine the cells.

### 2.13. Statistical Analysis

Statistical analysis was conducted using SPSS 24.0 (IBM Corp., Armonk, NY, USA). Data were presented as the mean ± standard deviation (SD), unless otherwise stated. The χ^2^ or Fisher’s exact test was applied to determine the statistical significance of the relationship between MAGED2 protein expression and clinicopathological parameters. One-way ANOVA was performed for comparison between normal brain tissues (NBS) and different glioma groups (>2 groups) followed by a post-hoc test, including Bonferroni correction for multiple comparisons. Regression analysis was used to assess the prognostic significance of MAGED2 protein expression. Overall survival (OS) and recurrence-free survival (RFS) rates were calculated using the Kaplan–Meier technique, and the differences in the survival curves were analyzed using the log-rank test. During the multivariable regression analysis, prognostic variables were detected using Cox proportional hazard models. The final model includes variables with a *p* < 0.05. A statistically significant difference was defined as a difference of *p* < 0.05.

## 3. Results

### 3.1. TCGA Dataset and Oncomine Database MAGED2 mRNA and Protein Expression Analysis in Glioma

**Figure 1:**
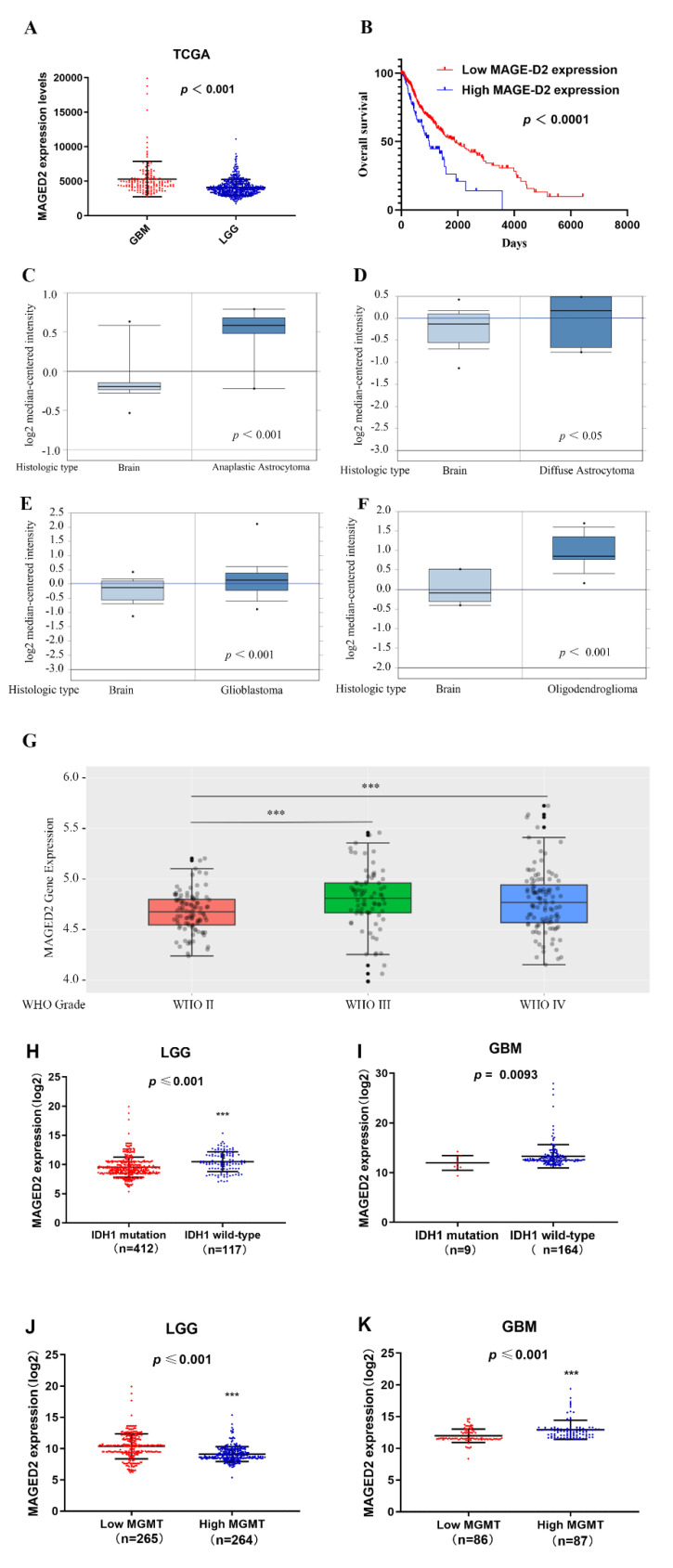
MAGED2 expression profiles and predictive value in glioma. (**A**) According to the TCGA dataset, MAGED2 expression levels are significantly higher in GBM tissues than in LGG tissues. (**B**) In glioma patients, higher MAGED2 expression is related to a shorter OS. (**C**–**F**) In the Oncomine database, MAGED2 expression in gliomas: (**C**) Anaplastic astrocytoma; (**D**) Diffuse astrocytoma; (**E**) GBM; and (**F**) Oligodendroglioma. (**G**) In the CGGA database, MAGED2 expression was found in gliomas of WHO grades II–IV. Horizontal lines represent the 25th-, 50th-, and 75th-percentile values; whiskers represent the 10th- and 90th-percentile values; and dots represent the maximum and minimum values. (**H**,**I**): the TCGA database was used to investigate the relationship between MAGED2 expression and IDH1 mutation in LGG and GBM. (**J**,**K**): the TCGA database was used to investigate the relationship between MAGED2 expression and MGMT expression in LGG and GBM.Data are represented as mean ± SD. *** *p* < 0.001.

MAGED2 expression levels have been shown to be considerably greater in GBM tissues than in LGG tissues, according to the TCGA dataset (*t* = 8.21, *p* < 0.001, Figure 1A). Furthermore, the clinical data and gene expression profiles of the 152 GBM patients were matched with those of the 510 LGG patients to determine if MAGED2 expression levels were related to patient survival, which was accomplished using Kaplan–Maier analysis and log-rank comparison. The results are shown in Figure 1B, which shows that a greater MAGED2 expression level is associated with a shorter OS in glioma patients (*t* = 6.32, *p* < 0.010). These data suggested that a low MAGED2 expression level in glioma may be responsible for the patients’ good OS.

Furthermore, by examining the Oncomine microarray datasets, researchers may detect variations in MAGED2 mRNA expression in distinct types of gliomas. The Beroukhim and Sun Brain dataset query results showed that MAGED2 mRNA expression in different types of glioma tissues, such as anaplastic astrocytoma (*t* = 3.827, *p* < 0.001, Figure 1C), glioblastoma (*t* = 3.223, *p* < 0.001, Figure 1E), and oligodendroglioma (*t* = 2.722, *p* = 0.004, Figure 1F), were all significantly increased when compared with normal brain tissue, with the exception of diffuse astrocytoma (*t* = 0.915, *p* = 0.193, Figure 1D). Moreover, the CGGA database results revealed that the expression of MAGED2 increased dramatically as the grade of glioma increased (Figure 1G). Additionally, the data from the UCSC Xena browser showed that LGG patients with wild-type IDH1 and low MGMT expression had considerably greater MAGED2 expression (Figure 1J). However, there is no significant difference in MAGED2 expression between the wild-type and mutant IDH1 groups in GBM patients, perhaps because there are fewer patients with an IDH1 mutant (Figure 1I). Furthermore, MAGED2 expression was noticeably greater in the group with high MGMT expression compared to the group with low MGMT expression in IDH1 (Figure 1K).

The association of glioma grading with IDH1/2, 1p/19q, MGMT, Ki67, and MAGED2 gene expression is demonstrated in Table 2. There was a significant association between glioma grading and IDH1/2 (*p* < 0.05), 1p/19q (*p* < 0.05), MGMT (*p* < 0.05), Ki67 (*p* < 0.01), and MAGED2 (*p* < 0.01). By contrast, glioma grading was not associated with TERT (*p* = 0.156) and EGFR variant III (EGFRvIII) (*p* = 0.090) (Table 2).

In addition, utilizing the Human Protein Atlas (HPA) Database (http://www.proteinatlas.org (accessed on 20 June 2022)), the two sets of chips were HPA031572 and HPA031573, with a total of 24 cases of tissue. MAGED2 protein was found in 24 cases of glioma but not in normal tissue. According to the HPA database, the overall proportion of positive cells for MAGED2 protein in glioma was 100%. However, the expression intensity of MAGED2 protein is differential in gliomas. This included 25.0% with strong expression, 70.83% with moderate expression, and 4.17% with weak expression. The MAGED2 protein staining was primarily located in the cell cytoplasm and nucleus. These findings also revealed that MAGED2 may play a role in glioma formation, which may hold promise as a prognostic marker.

### 3.2. MAGED2 Was Overexpressed in Human Glioma Tissues

**Figure 2:**
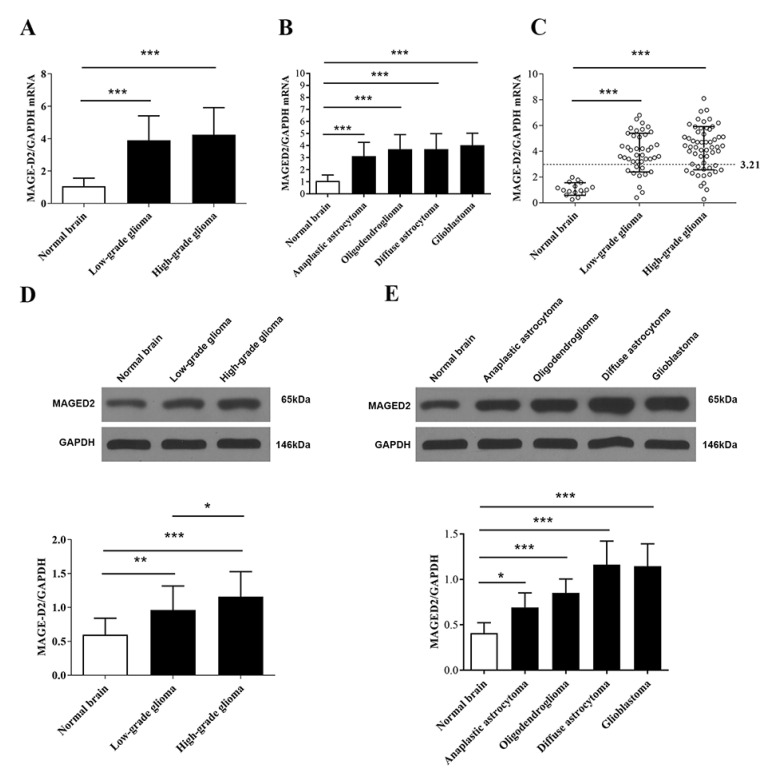
Levels of MAGED2 mRNA and protein expression in glioma versus healthy brain. (**A**,**D**) The median values of MAGED2 mRNA and protein expression in low-grade glioma and high-grade glioma were significantly higher than that of normal brain tissue. (**B**,**E**) MAGED2 mRNA and protein expression in several forms of gliomas revealed that glioblastoma had the greatest expression and diffuse astrocytoma had the second-highest expression, followed by oligodendroglioma and anaplastic astrocytoma. (**C**) MAGED2 mRNA high expression was determined to be three times higher than the median value of MAGED2/GAPDH in normal brain tissues. For high MAGED2 expression above the line, the cutoff value is 3.21. The percentage of low-grade (WHO, I–II) and high-grade (WHO, III–IV) gliomas with high MAGED2 mRNA expression was compared to that of normal brain tissue. Values are presented as the mean ± SD. The error bars represent the SD. (* *p* < 0.05, ** *p* < 0.01 and *** *p* < 0.001 when compared with normal brain tissue; χ^2^ test with subsequent Bonferroni’s correction).

The expression levels of MAGED2 were analyzed in 98 glioma and 16 normal brain tissue specimens using RT-qPCR and Western blotting. There were significant differences between low- or high-grade glioma compared with normal brain tissues for MAGED2 mRNA levels (Figure 2A, *p* < 0.001). MAGED2 mRNA was considerably greater in low-grade glioma (*p* < 0.001) and high-grade glioma (*p* < 0.001) than in normal brain tissues, but there was no significant difference in MAGED2 mRNA between high-grade and low-grade gliomas (Figure 2A). One-way ANOVA revealed that there are no significant differences in MAGED2 RNA expression between different types of gliomas, but it is significantly higher compared to healthy brain tissue (Figure 2B, *p* < 0.001). MAGED2 mRNA was highest in glioblastoma (*p* < 0.001), and diffuse astrocytoma had the second-highest value (*p* < 0.001)—followed by oligodendroglioma (*p* < 0.001) and anaplastic astrocytoma (*p* < 0.001)—when compared with normal brain tissue. High expression of MAGED2 was defined as a three-fold increase in the median value of MAGED2/GAPDH in normal brain tissues, as shown in Figure 2C. The findings revealed that 57.14% of glioma tissues demonstrated high mRNA MAGED2 expression, whereas 0% of normal brain tissues did (*p* < 0.001). When the percentage of high mRNA MAGED2 expression in low-grade (20.41%) and high-grade (36.73%) gliomas was compared to that of normal brain tissue, significant differences were found (*p* < 0.001 and *p* < 0.001, respectively); however, no significant differences were found between high-grade and low-grade glioma (*p* = 0.82; Figure 2C). Meanwhile, MAGED2 protein expression follows the same pattern as mRNA expression (*p* < 0.001, Figure 2D,E).

### 3.3. IHC to Examine the Expression of MAGED2 Protein

**Figure 3:**
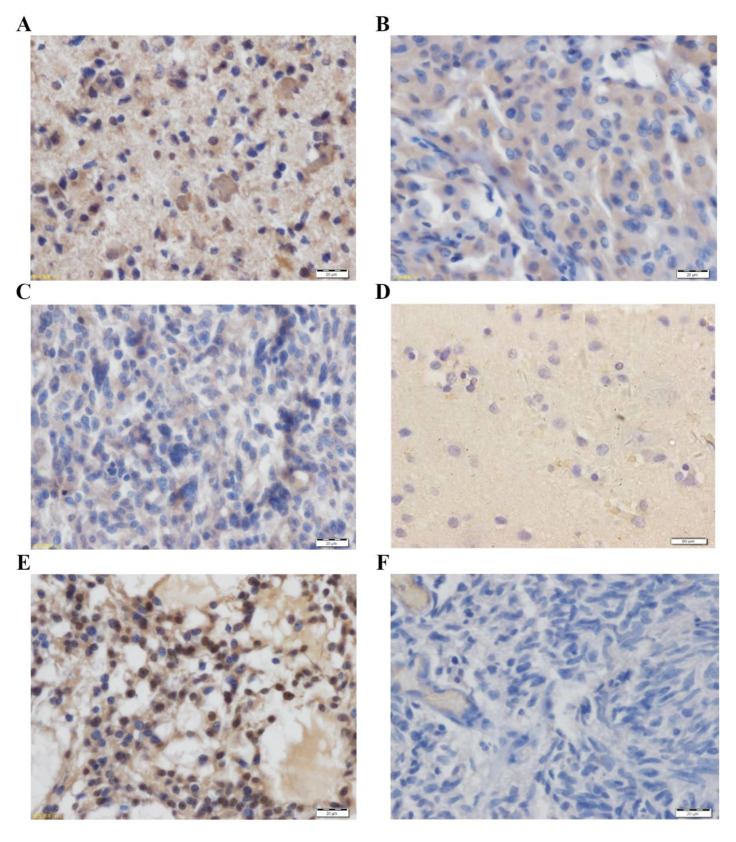
MAGED2 protein immunohistochemistry in (**A**–**C**) glioma tissues and (**D**) normal brain tissue. The staining was concentrated in the cytoplasm and nucleus of the cells. Images (**A**–**C**) show strong, moderate, and mild immunoreactivities with the polyclonal MAGED2 antibody, respectively. (**D**) In normal brain tissue, MAGED2 protein staining was negative. (**E**) A known glioma tissue segment with MAGED2-positive expression was used as a positive control. (**F**) Omission of the primary antibody was used as a negative control. Scale bar: 20 μm.

IHC was used to examine MAGED2 protein expression in 98 glioma tissues (Table 3). The overall proportion of cells that were positive for MAGED2 protein was 79.59%, according to the findings. The MAGED2 protein was mostly stained in the cytoplasm and nucleus of the cells. The percentage of cells in low-grade glioma samples that expressed MAGED2-positive protein was 30.61%, whereas it was 48.97% in high-grade gliomas (Table 3). According to the staining intensity, 57.14% of patients had high MAGED2 protein expression (Figure 3A,B), whereas 42.86% had low MAGED2 protein expression (Figure 3C). Furthermore, the presence of MAGED2 was essentially non-existent in 12 normal brain tissues that were stained (Figure 3D). The positive control was a known glioma tissue segment with MAGED2-positive expression (Figure 3E), while the negative control was the omission of the primary antibody (Figure 3F).

### 3.4. Clinicopathological Characteristics and MAGED2 Protein Expression

Table 3 and Supplementary Table S1 show the relationship between MAGED2 protein expression and clinical variables. MAGED2 protein expression was shown to be associated with WHO grade (*p* < 0.01), Ki-67 (*p* < 0.01), IDH1/2 (*p* < 0.05), and MGMT (*p* < 0.05). Conversely, MAGED2 protein expression was not associated with sex (*p* = 0.765), age (*p* = 0.814), tumor size (*p* = 0.860), or extent of resection (*p* = 0.554) (Table 3 and Supplementary Table S1).

### 3.5. Prognostic Impact of MAGED2 Protein Overexpression

**Figure 4:**
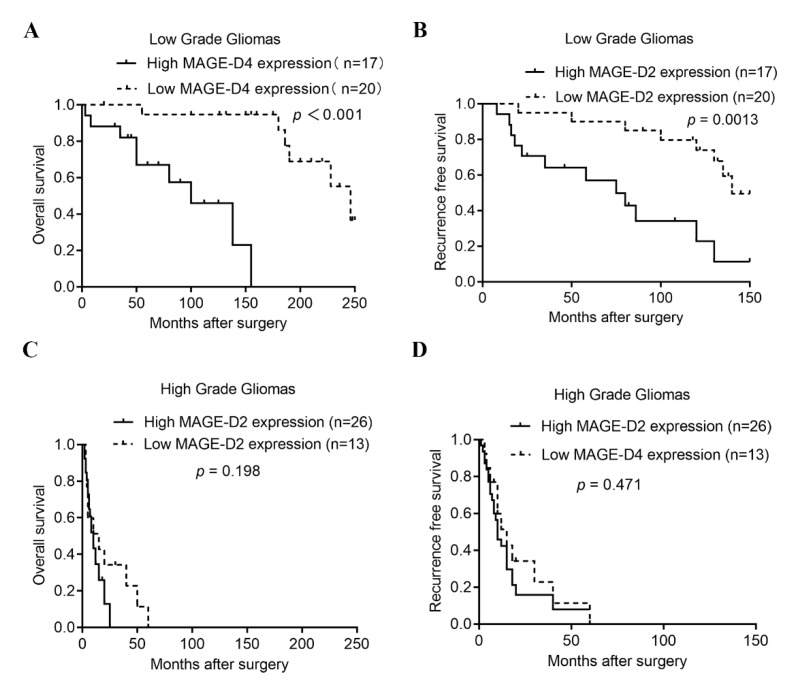
Kaplan–Meier curves showing the survival of glioma patients with different MAGED2 expression levels. (**A**–**D**) Kaplan–Meier survival curves plotting OS and RFS times according to MAGED2 staining in low-grade (**A**,**B**) and high-grade gliomas (**C**,**D**). Mantel–Cox log rank test was performed to determine the *p*-value indicated on the graphs. RFS, recurrence-free survival; OS, overall survival.

The influence of MAGED2 expression and tumor categorization was analyzed using a Kaplan–Meier survival analysis to identify the prognostic value for MAGED2. In the current investigation, 98 patients with glioma were followed up on and had complete clinical data. Patient follow-up lasted an average of 27.4 months (range: 19–72 months). In patients with glioma, a substantial positive correlation between MAGED2 protein expression, OS, and RFS times were discovered using clinical records. Patients with high MAGED2 protein expression had significantly shorter median OS (100.00 vs. 246.00 months; *p* < 0.001) and RFS (75.00 vs. 140.00 months; *p* = 0.0013) times in low-grade gliomas (Figure 4A,B); however, there was no significant difference in OS and RFS times in high-grade gliomas (*p* > 0.05; Figure 4C,D). Sex, age, tumor size (≥3.0 cm), extent of resection, WHO classification (High grade), IDH1 status (Mutant), Ki-67 expression (≥10%), and MAGED2 protein expression were used to categorize patients. Among these variables, an age of 39 years or older, WHO classification of high grade, IDH1 status (Mutant), Ki-67 expression ≥ 10%, and high MAGED2 protein expression were all found to be important prognostic indicators in OS and RFS using univariate analysis. Patient survival was influenced by a variety of factors, necessitating the use of a multivariate analysis. The WHO classification, IDH1 status (Mutant), and MAGED2 protein expression were all found to have a substantial influence on OS and RFS in patients with glioma. Since there was a substantial positive relationship between high MAGED2 protein expression and glioma prognosis, it was assumed that MAGED2 protein expression may be identified as an independent prognostic factor for glioma patients (Table 4).

### 3.6. MAGED2 Knockdown in Human Glioma U251-MG Cells Using MAGED2 CRISPR

**Figure 5:**
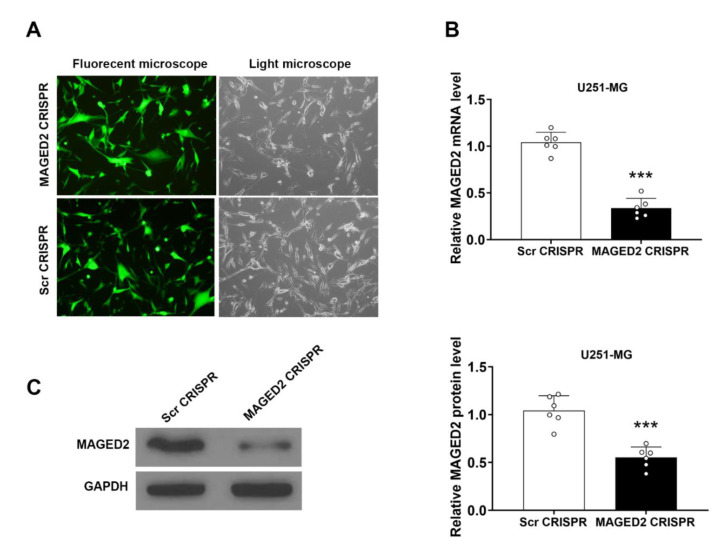
The glioma U251-MG cells were transfected with a lipofectamine vector expressing the MAGED2 CRISPR. (**A**) Fluorescence microscopy is used to assess transfection effectiveness 72 h after transfection. qRT-PCR test is used to determine the knockdown effectiveness of MAGED2 (**B**) and Western blotting (**C**). *** *p* < 0.001 compared with the Scramble CRISPR group; *n* = 6/group.

The human glioma U251-MG cell line was one of the most widely used glioma cell lines, with high tumorigenicity in vivo. The GFP-expressing MAGED2 CRISPR and Scramble CRISPR were created and transfected into U251-MG cells to better understand the role of MAGED2 in glioma. After lipofectamine transfection, almost 90% of the cells displayed positive green fluorescence, as indicated in the data (Figure 5A). qRT-PCR and Western blotting were used to determine the knockdown effectiveness. MAGED2 mRNA (*t* = 4.47, *p* = 0.0012, Figure 5B) and protein (*t* = 10.58, *p* < 0.000, Figure 5C) levels in U251-MG cells of the MAGED2 CRISPR group were significantly lower than those in the Scramble CRISPR group 72 h after infection.

### 3.7. MAGED2 Knockdown Significantly Reduced Glioma U251-MG Cell Growth

**Figure 6:**
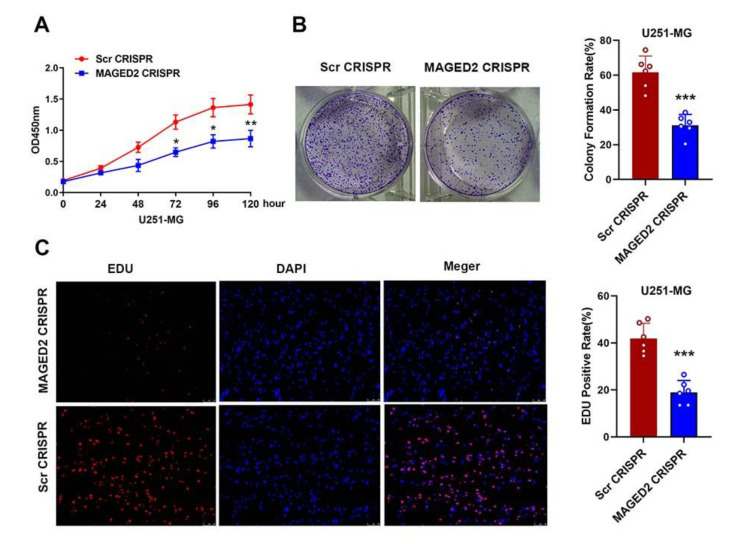
Glioma cell proliferation and colony formation are inhibited when MAGED2 is downregulated. (**A**) MAGED2 inhibition decreases the growth of glioma U251-MG cells in CCK-8 tests. (**B**) Colony formation also showed that the proliferation of U251-MG cells significantly reduced when MAGED2 is inhibited. (**C**) EdU tests indicate that downregulation of MAGED2 inhibits the proliferation of glioma U251-MG cells. * *p* < 0.05, ** *p* < 0.01, *** *p* < 0.001 compared with the Scramble CRISPR group; *n* = 6/group.

CCK-8 and colony formation experiments were performed on glioma U251-MG cells transfected with MAGED2 CRISPR or Scramble CRISPR to better understand the role of MAGED2 in glioma cell proliferation. As shown in Figure 6, MAGED2 knockdown significantly reduced the proliferation capacity and growth of U251-MG cells processed with MAGED2 CRISPR compared to the Scramble CRISPR group. Furthermore, the in vitro proliferation of the MAGED2 CRISPR-treated cells was significantly reduced at 72 h (Scr CRISPR: 1.13 ± 0.19 vs. MAGED2 CRISPR: 0.65 ± 0.12, *t* = 3.65, *p* = 0.02), 96 h (Scr CRISPR: 1.36 ± 0.16 vs. MAGED2 CRISPR: 0.80 ± 0.16, *t* = 4.34, *p* = 0.01), and 120 h (Scr CRISPR: 1.41 ± 0.17 vs. MAGED2 CRISPR: 0.86 ± 0.12, *t* = 4.62, *p* < 0.01) (Figure 6A). Similarly, the inhibition of MAGED2 also resulted in a significant reduction in colony formation (*t* = 5.22, *p* < 0.001, Figure 6B). The effect of MAGED2 on proliferation following MAGED2 downregulation was also assessed using the EDU cell-image assay. The results of the EDU cell-image test were consistent with the results of the CCK-8 and colony formation assays, indicating that the EdU-positive rates of U251-MG cells were lower in the MAGED2 CRISPR group than in the Scramble CRISPR group (*t* = 5.47, *p* < 0.0001, Figure 6C). As a result of these three tests, it was concluded that knocking-down MAGED2 inhibited the proliferation of glioma U251-MG cells.

### 3.8. Glioma U251-MG Cells Were Arrested in G0/G1 Phase and Apoptosis Was Accelerated by MAGED2 Suppression

**Figure 7:**
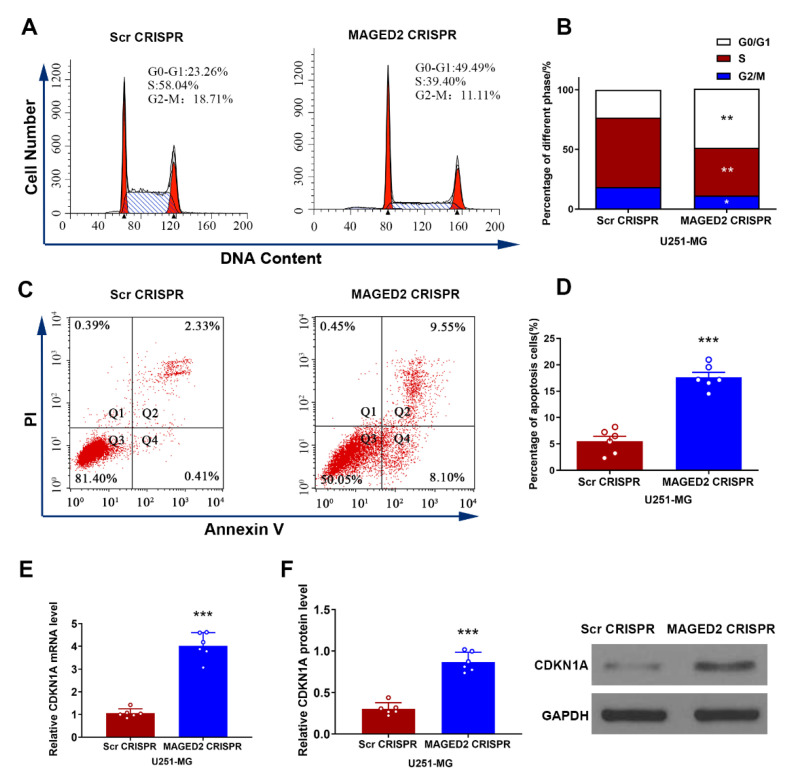
In glioma U251-MG cells, MAGED2 regulates the cell cycle, apoptosis, and CDKN1A expression levels. (**A**–**D**) Flow cytometry of the cell cycle distribution (**A**,**B**) and apoptosis (**C**,**D**) level of U251-MG cells transfected with MAGED2 CRISPR or Scramble CRISPR. (**E**–**F**) The mRNA (**E**) and protein (**F**) expression levels of CDKN1A detected by Western blotting and qRT-PCR after transfection with MAGED2 CRISPR or Scramble CRISPR. * *p* < 0.05, ** *p* < 0.01, and *** *p* < 0.001 compared with the Scramble CRISPR group; *n* = 6/group.

The cell cycle distribution demonstrated a direct relationship with cell proliferation. On this basis, flow cytometry was used to study the cell cycle distribution of U251-MG cells following MAGED2 downregulation. As depicted in Figure 7A,B, inhibiting MAGED2 increased the number of cells entering the G0/G1 phase by 26.23% (*t* = 10.51, *p* < 0.001), while decreasing the number of cells entering the S phase by 18.64% (*t* = 13.03, *p* < 0.001) and the number of cells entering the G2/M phase by 7.64% (*t* = 3.76, *p* = 0.019). Furthermore, the impact of MAGED2 knockdown on apoptosis in glioma U251-MG cells was also investigated. The results showed that the percentage of apoptosis in U251-MG cells from the MAGED2 CRISPR group (17.64% ± 2.44%) was significantly greater than the Scramble CRISPR group (5.50% ± 1.85%) (*t* = 9.17, *p* < 0.001, Figure 7C,D). These findings demonstrated that MAGED2 knockdown effectively inhibited U251-MG cell proliferation by increasing the proportion of cells in the G0/G1 phase, thereby lowering the percentage of cells in the S phase and triggering apoptosis.

### 3.9. MAGED2 Downregulation Decreased Cell Proliferation through Restoring CDKN1A

U251-MG cells were transfected in vitro. Following that, CDKN1A mRNA and protein expression levels were determined by qRT-PCR and Western blotting, respectively. The results showed that when compared to Scramble CRISPR, CDKN1A mRNA expression levels were clearly upregulated following transfection with MAGED2 CRISPR (*t* = 12.33, *p* < 0.0001, Figure 7E). Furthermore, CDKN1A protein expression was much greater in the MAGED2 CRISPR group (*t* = 11.52, *p* < 0.0001, Figure 7F).

### 3.10. Downregulation of MAGED2 Inhibited the Proliferation of U251-MG Cells In Vivo

**Figure 8:**
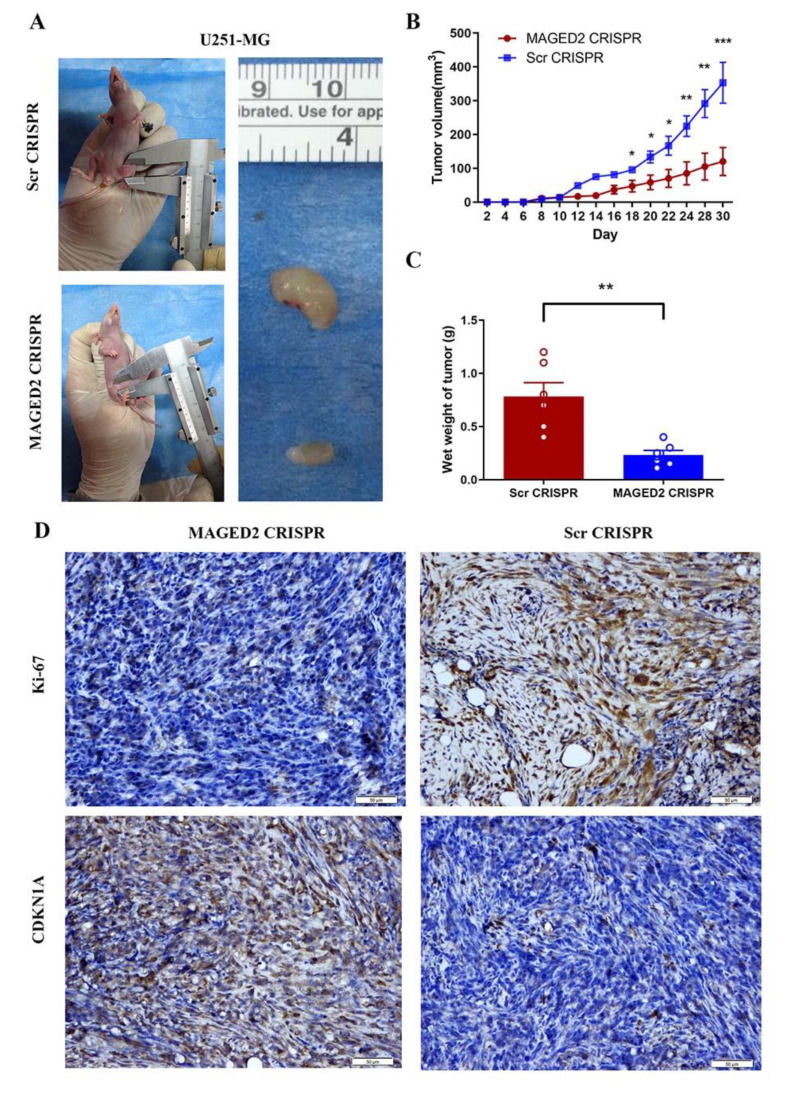
MAGED2 promoted glioma cell proliferation in vivo. The glioma cell growth of the U251-MG cell line was determined by xenograft model assay in vivo. U251-MG- MAGED2 CRISPR and Scramble CRISPR cells were transplanted subcutaneously into nude mice and monitored every 2 days (**A**). Tumor growth curves were determined (**B**), and tumor weight (**C**) was shown. The error bar represents the SD of the mean value. Statistical analysis was determined by two-tailed Student’s *t*-test. * *p* < 0.05, ** *p* < 0.01, *** *p* < 0.001; *n* = 6/group. (**D**) MAGED2 downregulation inhibits the growth of glioma cells in vivo. Ki-67 and CDKN1A in tumors are assessed in U87 cells by IHC assay after treatment with MAGED2 CRISPR and Scramble CRISPR. Scale bar: 50 μm.

In vitro, knocking-down MAGED2 would stop glioma cells from proliferating. To see if inhibiting MAGED2 decreased the proliferation of glioma in vivo, human U251-MG cells were subcutaneously implanted into the bilateral flanks of immunodeficient athymic Balb/c nude mice (4 weeks) using MAGED2 CRISPR or Scramble CRISPR steadily transfected cells. When compared to the Scramble CRISPR group, the tumor volume in the MAGED2 CRISPR group was significantly reduced. Overall, these data demonstrated that MAGED2 plays a key role in glioma growth and expansion in vivo (Figure 8A–C). In addition, IHC tests were used to investigate the levels of Ki-67, which was previously only used to predict tumor growth. In the MAGED2 CRISPR group, there was a substantial drop in Ki-67 proteins (Figure 8D). Furthermore, IHC data demonstrated upregulated CDKN1A expression in the MAGED2 CRISPR group, which matched the in vitro results (Figure 8D). Finally, our findings showed that downregulating MAGED2 in vivo will decrease glioma tumor growth by increasing CDKN1A expression.

### 3.11. Downregulation of CDKN1A Expression Restores Glioma Cell Proliferation in U251-MG Cells

**Figure 9:**
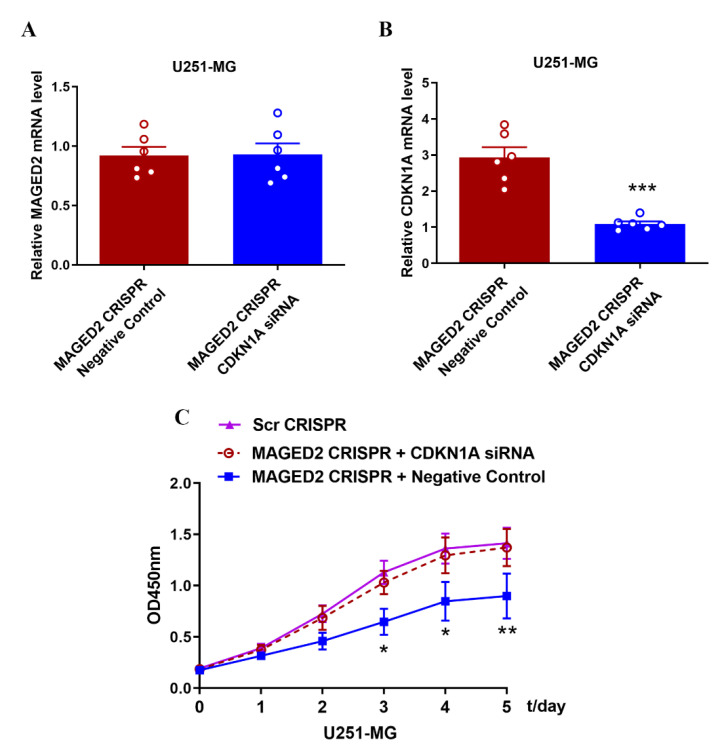
Downregulation of CDKN1A expression resumes the proliferation of glioma U251-MG cells. (**A**,**B**) After transfection of CDKN1A siRNA in MAGED2 CRISPR U251-MG cells, MAGED2 (**A**) and CDKN1A (**B**) mRNA expression were detected by qRT-PCR, * *p* < 0.05, ** *p* < 0.01, and *** *p* < 0.001; *n* = 6/group, and the CCK-8 assay (**C**) was used to detect the proliferative capacity of glioma U251-MG cells.

After MAGED2 CRISPR U251MG cells were transfected with CDKN1A siRNA, the relative levels of MAGED2 expression in the CDKN1A siRNA group did not substantially change from those in the negative control group (a fluorescent siRNA with no homology to target gene sequence) (Figure 9A); however, the relative CDKN1A mRNA expression levels differed considerably from those in the negative control group (Figure 9B). Furthermore, downregulating CDKN1A expression partially restored the capacity of U251MG cells to proliferate, which had been reduced by the MAGED2 CRISPR (Figure 9C).

## 4. Discussion

The discovery of useful prognostic biomarkers will aid in early diagnosis, tumor therapy response prediction, prognosis, and, eventually, personalized treatment. The most recent version of the WHO Classification for 2021 was published, providing more accurate stratification than classification based solely on histopathology [21]. Indeed, it pioneered molecular indicators such as IDH1/2 mutation status and 1p/19q co-deletion, which are now well acknowledged for their excellent predictive value across the world [21]. Despite the long history of molecular research into the glioma profile, MAGED2 has never been published to our knowledge, even though we have established that it is an intriguing prognostic marker.

Many malignancies have high MAGED2 expression, and MAGED2 may play a vital role in cancer progression, making it a prospective target for tumor therapy. MAGED2 mRNA overexpression leads to tumor growth in hepatocellular carcinoma, according to Kanda and colleagues, and thus may serve as a prognosis indicator after curative resection as well as a possible therapeutic target in hepatocellular carcinoma [16].

Further studies have confirmed that MAGED2 expression levels were associated with the metastatic potential of gastric cancer, indicating that patients with gastric cancer had a poor prognosis. Such findings suggest that MAGED2 could be a viable biomarker for malignant gastric cancer behavior in both gastric tissue and serum samples [17]. Type-2 MAGE expression has recently been linked to crucial clinical and molecular aspects in glioma, according to research [9,10]. However, in a bioinformatics study, MAGED2 did not show a distinct pattern of overexpression/downregulation [12]. As a result, the precise involvement of MAGED2 in the progression and prognosis of glioma patients remains unknown.

The public expression profiles and clinical data of glioma patients from the TCGA were first used in the current investigation. According to the TCGA data, MAGED2 expression levels were significantly higher in GBM tissues than in LGG tissues. Furthermore, as compared to NBTs, MAGED2 was shown to be substantially expressed in human glioma tissues. In the CGGA database, the expression of MAGED2 rose dramatically as the grade of glioma increased. IDH1 mutation and MGMT expression were both linked to MAGED2 expression. Secondly, a statistical analysis revealed that patients with high MAGED2 expression in malignant tissues had significantly shorter median OS and RFS times than those with low MAGED2 expression, according to a Kaplan–Meier study. MAGED2 could be used as an independent predictive biomarker in glioma patients, according to multivariate and univariate survival studies. Thirdly, a MAGED2 CRISPR KO-expressing lipofectamine vector was created and transfected into glioma U251-MG cells, allowing it to stably downregulate MAGED2’s expression levels in vitro. The findings suggested that MAGED2 knockdown could inhibit U251-MG cell growth by raising the percentage of cells in the G0/G1 phase while decreasing the percentage of cells in the S phase, and by triggering apoptosis.

Papageorgio et al. recently showed that MAGED2 could boost lung cancer cell proliferation by targeting CDKN1A in lung cancer. Additionally, in certain malignancies, MAGED2, like NDN, may promote p53-mediated growth inhibition via a CDKN1A-independent mechanism [14]. Furthermore, when compared to Scramble CRISPR, this study found that mRNA and protein expression levels of CDKN1A were significantly upregulated after transfection with MAGED2 CRISPR in vitro. CDKN1A is a cyclin-dependent kinase inhibitor that stops the cell cycle by inhibiting the CDK1 and CDK2 complexes [30].

The downregulation of MAGED2 expression inhibited the growth of glioma cells in our investigation. However, our findings show that MAGED2 expression was related to the G1 phase of the cell cycle rather than the G2/M phase. This could be due to the varied cells that were selected. We also discovered that MAGED2 regulates glioma U251-MG cell growth via CDKN1A. CDKN1A has been demonstrated to influence cell cycle progression in the S and G1 phases in numerous earlier investigations [31]. It has been confirmed that CDKN1A interacts with the proliferating cell nuclear antigen (PCNA), which is involved in S-phase DNA replication and DNA damage repair [32]. As a result, the MAGED2/CDKN1A pathway may be related to the G1 phase of the cell cycle in glioma U251-MG cells.

Finally, downregulating CDKN1A expression partially restored the capacity of U251MG cells to proliferate, which had been reduced by the MAGED2 CRISPR. The tumor suppressor protein, p53, regulates CDKN1A expression, and CDKN1A can induce p53-dependent cell cycle arrest at G1 in response to a variety of stressors [32]. Trussart et al. discovered that melanoma antigen D2 regulates cell cycle progression and the DNA damage response in a variety of cancers [33]. According to Papageorgio et al., MAGED2 is a possible negative regulator of p53 activity, which could have consequences for cancer management and prognosis [14]. As a result, we hypothesize that MAGED2’s function is partially dependent on the CDKN1A/p53 pathway. However, whether other key elements are also involved in MAGED2’s regulatory network in glioma warrants additional investigation. As a result, the data suggest that MAGED2 may increase the proliferation of glioma U251-MG cells by inhibiting CDKN1A.

The current study included several limitations. Firstly, the goal of this study was to determine the changes in MAGED2 expression between glioma and normal brain tissue, as well as the impact of MAGED2 on the prognosis of glioma patients. Future in vitro and in vivo investigations should use MAGED2-positive and -negative glioma cells to determine the biological function of MAGED2. Secondly, glioma progression involves a complicated network of pathophysiology. The pathway of MAGED2-mediated cell cycle progression also involves ATR, SKP2, and CDC20 signaling to a lesser extent. Finally, because the current study was conducted in a single institute, there should be more patients included in the study, and the patient classification should be balanced. It is also critical to detect serum immunoreactivity in glioma patients. The ultimate goal of the experimental line of inquiry is to lay the groundwork for MAGED2-based immunotherapy.

## 5. Conclusions

In conclusion, the current study found that MAGED2 plays an important role in the prognosis and growth of human glioma. MAGED2 is often overexpressed in human glioma tissues, which predicts a poor prognosis for glioma patients. Furthermore, the downregulation of MAGED2 expression by MAGED2 CRISPR inhibited the proliferation of glioma U251-MG cells in vitro by upregulating CDKN1A. Furthermore, these findings suggest that MAGED2 can stimulate the growth of U251-MG cells by targeting CDKN1A, suggesting that MAGED2 may serve as a novel target in the clinical treatment of glioma.

## Figures and Tables

**Table 1:** Characteristics of patients with gliomas.

Characteristic	Numbers *n* = 98	(%)
Gender		
Male	59	60.20
Female	39	39.79
Age (years)		
<39	47	47.96
≥39	51	52.04
Tumor size (cm)		
<3	31	31.63
≥3	67	68.36
Extent of resection		
Total resection	63	64.29
Subtotal resection	35	35.71
Tumor side		
Right	56	57.14
Left	42	42.86
Infiltrated part		
Single	36	36.73
Multiple	62	63.27
WHO classification ^a^		
Low	40	40.81
High	58	59.18
KPS		
<70	37	37.76
≥70	61	62.24
Treatment after first surgery		
RT	6	6.12
Chemo	21	21.43
RT + Chemo	58	59.18
None	13	13.27

^a^ According to the 2021 World Health Organization Classification of Tumors of the Nervous System. WHO, World Health Organization; KPS, Karnofsky Performance Scale.

**Table 2:** Summary of molecular data of samples of included gliomas (*n* = 98).

Variable	Lower Grade Gliomas	Higher Grade Gliomas	χ^2^	*p*-Value
IDH1/2				
Mutant	22.45%	30.61%	6.184	0.013 *
Wild-type	31.63%	15.31%		
1p/19q				
Co-deletion	19.39%	34.69%	4.713	0.029 *
Non-co-deletion	26.53%	19.39%		
TERT				
Positive	26.53%	23.47%	2.013	0.156
Negative	19.39%	30.61%		
MGMT				
Methylated	14.28%	29.59%	5.507	0.019 *
Unmethylated	31.63%	24.49%		
EGFRvIII				
Positive	23.47%	36.73%	2.872	0.090
Negative	22.45%	17.35%		
Ki67				
<10%	30.61%	20.41%	8.151	0.004 *
≥10%	15.31%	33.67%		
MAGE-D2				
Positive	17.35%	37.67%	10.09	0.002 **
Negative	28.57%	16.33%		

* *p* < 0.05, ** *p* < 0.01.

**Table 3:** Association between the MAGED2 protein and clinical characteristic of glioma patients (*n* = 98).

Characteristics	Positive/Total Test (%)	Positive/Total (%)		χ^2^	*p*
		High ^a^	Low ^b^		
Gender				0.089	0.765
Male	44/98 (44.89)	35/98 (35.71)	25/98 (25.51)		
Female	34/98 (34.69)	21/98 (21.43)	17/98 (17.35)		
Age (years)				0.055	0.814
<39	40/98 (40.81)	27/98 (24.49)	19/98 (19.39)		
≥39	38/98 (38.78)	32/98 (32.65)	20/98 (23.47)		
Tumor size (cm)				0.031	0.860
<3	39/98 (39.71)	25/98 (25.51)	18/98 (18.37)		
≥3	39/98 (39.71)	31/98 (31.63)	24/98 (24.49)		
Extent of resection				0.350	0.554
Total resection	43/98 (43.88)	34/98 (34.69)	23/98 (23.47)		
Subtotal resection	35/98 (35.71)	22/98 (22.45)	19/98 (19.39)		
WHO grade				7.850	0.005 **
Low	30/98 (30.61)	20/98 (20.41)	27/98 (27.55)		
High	48/98 (48.97)	36/98 (36.73)	15/98 (15.31)		
Total	78/98 (79.59)	56/98 (57.14)	42/98 (42.86)	-	-

^a^ High MAGED2 protein expression (++/+++); ^b^ Low MAGED2 protein expression (−/+). ** *p* < 0.01.

**Table 4:** Univariate and Multivariate analysis of different prognostic parameters.

All Gliomas Variable		Overall Survival				Recurrence-Free Survival			
		Univariate Analysis ^a^ Hazard Ratio (95% CI) ^c^	*p*-Value	Multivariate Analysis ^b^ Hazard Ratio (95% CI) ^c^	*p*-Value	Univariate Analysis ^a^ Hazard Ratio (95% CI) ^c^	*p*-Value	Multivariate Analysis ^b^ Hazard Ratio (95% CI) ^c^	*p*-Value
Gender	Male	0.644 (0.452–1.102)	0.169			0.764 (0.415–1.202)	0.309		
Age	≥39 years	1.061 (1.030–1.089)	0.018 ^d^	0.964 (0.902–1.031)	0.965	1.064 (1.022–1.083)	0.009 ^d^	1.003 (0.832–1.042)	0.368
Tumor size	≥3 cm	1.129 (0.593–1.953)	0.969			1.079 (0.436–1.672)	0.089	-	-
Extent of resection	Total resection	1.562 (0.724–2.362)	0.235			1.6889(0.944–2.687)	0.069		
WHO grade	High grade	3.595 (1.544–5.571)	<0.001 ^d^	3.556 (2.952–6.853)	<0.001 ^d^	3.148 (1.533–5.770)	<0.001 ^d^	3.048 (1.519–5.453)	<0.001 ^d^
IDH1 status	Mutant	18.553 (10.844–38.197)	<0.001 ^d^	12.877 (6.053–23.167)	<0.001 ^d^	10.445 (4.347–13.277)	<0.001 ^d^	8.756 (3.649–12.486)	<0.001 ^d^
Ki-67	≥10%	6.179 (2.168–15.411)	<0.001 ^d^	2.766 (1.068–5.437)	0.575	9.474 (3.168–26.771)	<0.001 ^d^	5.476 (1.705–12.656)	0.156
MAGE-D2	Positive	0.578 (0.182–0.927)	0.019 ^d^	1.271 (0.502–2.327)	0.782	0.471 (0.102–0.827)	0.009 ^d^	0.984 (0.508–1.947)	0.075

^a^ Univariate analysis was performed using the log-rank test. ^b^ Multivariate analysis was performed using the Cox proportional hazards model. ^c^ HR, Harzard ratio; 95 percent CI, 95 percent confidence interval for relative risk. ^d^ Statistically significant (*p* < 0.05).

## Data Availability

The datasets analyzed during the current study are available from the corresponding author on reasonable request.

## Supplementary Materials

The following supporting information can be downloaded at: https://www.mdpi.com/article/10.3390/brainsci12080986/s1, Table S1: Association between the MAGE-D2 protein and clinical characteristic of glioma patients (*n* = 98).

## Abbreviations

MAGE, melanoma-associated antigen; IHC, immunohistochemistry; OS, overall survival; RFS, recurrence-free survival; TCGA, The Cancer Genome Atlas; NBS, normal brain tissues.
