# Supplementary material for: Expression and Prognostic Value of Melanoma-Associated Antigen D2 in Gliomas
Source: Brain Sci. 2022 Jul 26;12(8):986. doi: 10.3390/brainsci12080986 (PMC9330880; doi:10.3390/brainsci12080986)
Supplement: Supplementary file 1 [file brainsci-12-00986-s001.zip › brainsci-1758574-supplementary.pdf]

SUPPLEMENTAL MATERIAL

Supplemental Table

Table S1. Association between the MAGE-D2 protein and clinical characteristic of glioma patients (*n*=98)

Characteristics	Positive / Total test (%)	Positive / total（%）		χ^2	P
		High[a]	Low[b]		
Ki-67 (%)				6.908	0.008**
＜10%	40/98(40.81)	19/98(19.39)	26/98(26.53)		
≥10%	38/98(38.77)	37/98(37.76)	17/98(16.33)		
IDH1/2 (%)				5.479	0.019*
Mutant	30/98(30.61)	36/98(36.73)	17/98(17.35)		
wildtype	48/98(48.97)	20/98(20.41)	25/98(25.51)		
MGMT				4.901	0.027*
Methylated	33/98(33.67)	17/98(17.35)	27/98(27.55)		
Unmethylated	45/98(45.92)	33/98(33.67)	21/98(21.43)		
Total	78/98(79.59)	56/98(57.14)	42/98(42.86)	-	-

[a]: High MAGE-D2 protein expression (++/+++)；

[b]: Low MAGE-D2 protein expression (–/+);
